# 

**Published:** 1818-05

**Authors:** 


### CORRESPONDENCE.

Communications are received from Dr. Balfour, Dr. Boyd, Dr. Robertson, and Mr. Blackett.

*We have received a great number of announcements of Lectures, particularly from the different Medical Schools in Scotland, and would readily have inserted them, had we not been informed by the Commissioners of Stamps that every announcement would be charged with the advertisement duty; we shall therefore, in future, be under the necessity of charging five shillings for each Lecture, in order to defray the unavoidable expence of its insertion.*

